# Expanding the *FDXR*-Associated Disease Phenotype: Retinal Dystrophy Is a Recurrent Ocular Feature

**DOI:** 10.1167/iovs.62.6.2

**Published:** 2021-05-03

**Authors:** Neringa Jurkute, Priya D. Shanmugarajah, Marios Hadjivassiliou, Jenny Higgs, Miodrag Vojcic, Iain Horrocks, Yann Nadjar, Valerie Touitou, Guy Lenaers, Roy Poh, James Acheson, Anthony G. Robson, F. Lucy Raymond, Mary M. Reilly, Patrick Yu-Wai-Man, Anthony T. Moore, Andrew R. Webster, Gavin Arno, for the Genomics England Research Consortium

**Affiliations:** 1 Moorfields Eye Hospital NHS Foundation Trust, London, United Kingdom; 2 Institute of Ophthalmology, University College London, London, United Kingdom; 3 Academic Department of Neurosciences, Royal Hallamshire Hospital, Sheffield, United Kingdom; 4 Liverpool Centre for Genomic Medicine, Liverpool Women's Hospital, Liverpool, United Kingdom; 5 Departments of Neurology and Ophthalmology, Royal Victoria Infirmary, Newcastle upon Tyne, United Kingdom; 6 Paediatric Neurosciences Research Group, Royal Hospital for Children, Glasgow, Scotland; 7 Department of Neurology, Reference Center for Lysosomal Diseases, Neuro-Genetic and Metabolism Unit, Paris, France; 8 Sorbonne University, Paris, France; 9 Groupe Hospitalier La Pitié Salpêtrière-Charles Foix, DHU Vision Et Handicaps, Paris, France; 10 Université Angers, MitoLab team, UMR CNRS 6015 - INSERM U1083, Angers, France; 11 Department of Neurogenetics, The National Hospital for Neurology and Neurosurgery and UCL Institute of Neurology, London, United Kingdom; 12 National Hospital for Neurology and Neurosurgery, University College London Hospitals NHS trust, London, United Kingdom; 13 NIHR BioResource - Rare Diseases, Cambridge University Hospitals NHS Foundation Trust, Cambridge Biomedical Campus, Cambridge, United Kingdom; 14 Department of Medical Genetics, Cambridge Institute for Medical Research, University of Cambridge, Cambridge, United Kingdom; 15 Centre for Neuromuscular Diseases, UCL Queen Square Institute of Neurology and National Hospital for Neurology and Neurosurgery, London, United Kingdom; 16 Cambridge Eye Unit, Addenbrooke's Hospital, Cambridge University Hospitals, Cambridge, United Kingdom; 17 John van Geest Centre for Brain Repair and MRC Mitochondrial Biology Unit, Department of Clinical Neurosciences, University of Cambridge, Cambridge, United Kingdom; 18 Department of Ophthalmology, University of California, San Francisco, San Francisco, California, United States; 19 Great Ormond Street Hospital for Children, London, United Kingdom

**Keywords:** *FDXR*, ferredoxin reductase, syndromic optic neuropathy, retinal dystrophy, neurodegenerative disorder, iron accumulation

## Abstract

**Purpose:**

The purpose of this study was to report retinal dystrophy as a novel clinical feature and expand the ocular phenotype in patients harboring biallelic candidate *FDXR* variants.

**Methods:**

Patients carrying biallelic candidate *FDXR* variants were identified by whole genome sequencing (WGS) as part of the National Institute for Health Research BioResource rare-disease and the UK's 100,000 Genomes Project (100KGP) with an additional case identified by exome sequencing. Retrospective clinical data were collected from the medical records. Haplotype reconstruction was performed in families harboring the same missense variant.

**Results:**

Ten individuals from 8 unrelated families with biallelic candidate variants in *FDXR* were identified. In addition to bilateral optic atrophy and variable extra-ocular findings, 7 of 10 individuals manifested retinal dystrophy comprising dysfunction and degeneration of both rod and cone photoreceptors. Five of 10 subjects had sensorineural hearing loss. The previously unreported missense variant (c.1115C > A, p.(Pro372His)) was found in 5 of 8 (62.5%) study families. Haplotype reconstruction using WGS data demonstrated a likely ancestral haplotype.

**Conclusions:**

*FDXR*-associated disease is a phenotypically heterogeneous disorder with retinal dystrophy being a major clinical feature observed in this cohort. In addition, we hypothesize that a number of factors are likely to drive the pathogenesis of optic atrophy, retinal degeneration, and perhaps the associated systemic manifestations.

Biallelic variants of *FDXR*, encoding ferredoxin reductase, were recently reported to be associated with a variable mitochondrial disorder characterized by sensorineural hearing loss, visual impairment, and systemic, particularly neurological, manifestations.^1^^–^^5^ Since the first reports in 2017, a total of 35 cases have been reported worldwide.^1^^–^^5^

Ferredoxin reductase is a mitochondrial flavoprotein that is required for iron-sulfur cluster biogenesis, which are essential for a number of cellular processes, including electron transport along the mitochondrial respiratory chain, regulation of gene expression, substrate binding and activation, iron homeostasis, and DNA repair. Dysfunction or dysregulation of mitochondrial iron-sulfur cluster biosynthesis is known to lead to defects in iron homeostasis, iron overload, oxidative stress, and mitochondrial dysfunction.^6^^,^^7^

This study describes previously unreported candidate pathogenic variants in *FDXR* and details the association of *FDXR* variants with retinal dystrophy, further expanding the clinical phenotypic spectrum consequent upon *FDXR* defects.

## Materials and Methods

### Study Cohort

In this retrospective multicenter study, the clinical and genetic data of individuals with biallelic *FDXR* variants was reviewed to establish and expand the phenotypic spectrum of *FDXR*-associated disease. Interrogation of whole genome sequencing (WGS) data lead to the identification of nine families found to harbor candidate pathogenic *FDXR* genotypes. Three families were identified in the inherited eye disease clinics at Moorfields Eye Hospital NHS Foundation Trust (London, UK), with additional UK families identified via the UK's 100,000 Genomes Project (100KGP) recruited at University College London Hospitals NHS Foundation Trust (London, UK), the Newcastle upon Tyne Hospitals NHS Foundation Trust (Royal Victoria Infirmary, Newcastle upon Tyne, UK), the Liverpool Women's NHS Foundation trust (Liverpool, UK), and the Sheffield Teaching Hospitals NHS Foundation Trust (Royal Hallamshire Hospital, Sheffield, UK). In addition, one family was identified via exome sequencing of a cohort of French patients with bilateral optic atrophy, but no genetic diagnosis. This retrospective study adhered to the tenets of the Declaration of Helsinki and the contributing study centers had the relevant ethical and institutional approvals.

### Clinical Phenotyping

Medical records were reviewed for each individual following the identification of candidate pathogenic *FDXR* variants. In addition, all affected individuals underwent an ophthalmological examination during the initial diagnostic work up or following the molecular diagnosis.

Four individuals from three families (families 1–3) examined at Moorfields Eye Hospital underwent electrophysiological assessment. In the oldest child, pattern and full-field electroretinography (PERG; ERG) were performed according to international standards using gold foil corneal recording electrodes.^8^^,^^9^ The three youngest children were tested with gold foil electrodes (*N* = 1) or lower eyelid skin electrodes (*N* = 2) according to a shortened protocol.^10^ Flash visual evoked potential (VEP) testing was performed in the three youngest cases, including one that underwent additional pattern VEP testing to a large stimulus field to minimize the effects of fixation error.^11^

### Molecular Genetic Analysis

WGS was performed as part of the National Institute for Health Research BioResource rare-disease project (NIHR-RD) and 100KGP as previously described.^12^^,^^13^ To identify the most likely disease-causing variant, a multistep rare variant filtering pipeline was used. Family 1 (GC 21294) underwent rare variant filtering for protein altering variants in genes previously shown to be associated with inherited optic neuropathy, which did not identify any candidate variant/s.^12^ As autosomal recessive disease was suspected, the search was subsequently broadened to include potential biallelic protein altering variants across the entire genome with a minor allele frequency (MAF) < 0.001 (Genome Aggregation Database, gnomAD, https://gnomad.broadinstitute.org) which revealed two rare variants in *FDXR*. To identify additional individuals carrying biallelic variants in *FDXR*, a similar analysis was performed (focusing on the *FDXR* gene following exclusion of variants in known genes using a virtual gene panel [PanelApp]) in the NIHR-RD and 100KGP datasets from individuals with inherited ocular disorders.^14^ Exome sequencing was performed at BGI Genomics, China, followed by variant filtering for biallelic rare variants (MAF < 0.003). Pathogenic mtDNA variants were excluded in all individuals by direct whole mtDNA sequencing from blood or muscle tissue, or WGS. Additional rare (MAF < 0.001) variants within the optic neuropathy or retinal dystrophy gene panels not thought to contribute to disease are listed in Supplementary Table S1.

### Bioinformatics

To predict the functional impact of missense variants, in silico prediction tools were applied, including the predictive algorithms of Polymorphism Phenotyping version 2 (PolyPhen-2) and Mutation Taster predictive algorithms available at http://genetics.bwh.harvard.edu/pph2 and http://www.mutationtaster.org, respectively. The evolutionary conservation of the affected amino acid residues across orthologues was assessed using Uniprot sequence alignments (https://www.uniprot.org/help/sequence-alignments).

### Haplotype Analysis

The haplotypic background of the c.1115C>A, p.(Pro372His) variant was investigated using the WGS data from family 2 (GC 17577), family 4, family 6 (GC 28579), and family 7 (GC 28550). Single nucleotide variants (SNVs) up to approximately 150 kb up and downstream of the *FDXR* gene were phased to determine those in cis with the *FDXR* c.1115C>A variant. SNVs with MAF < 0.1 in the gnomAD dataset were considered to be informative for phasing. Hemizygous variants present in the affected individual (family 6, GC 28579) carrying c.1115C>A in trans with a large multigene deletion involving the entire *FDXR* gene, were assumed to be in cis with the c.1115C>A. Once these haplotypes and informative SNVs were established in four families, direct variant interrogation on WGS dataset was performed to identify the haplotype in the singleton from family 1.

## Results

**Figure 1.**
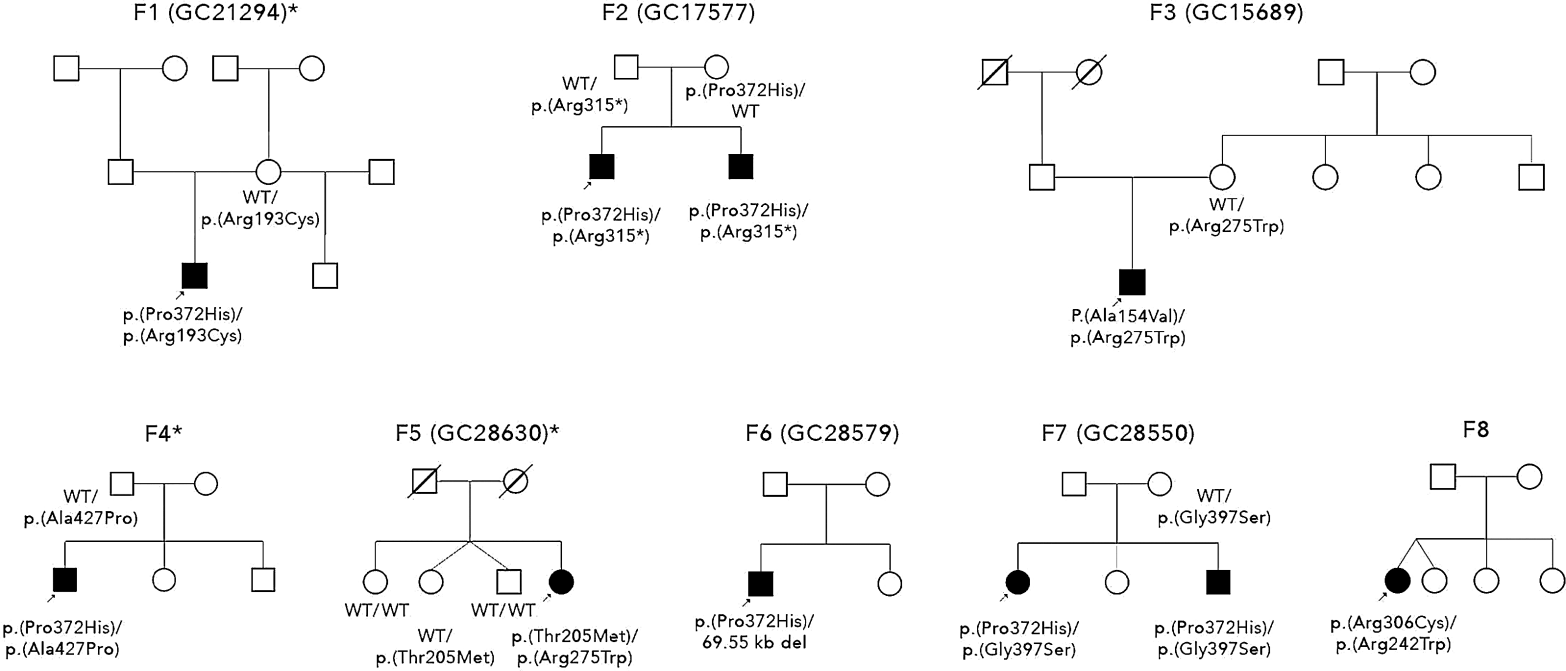
Pedigrees of families. An *arrow* indicates proband. Shaded shape represents affected individual. Asterisk (*) indicates families without retinal dystrophy.

**Figure 2.**
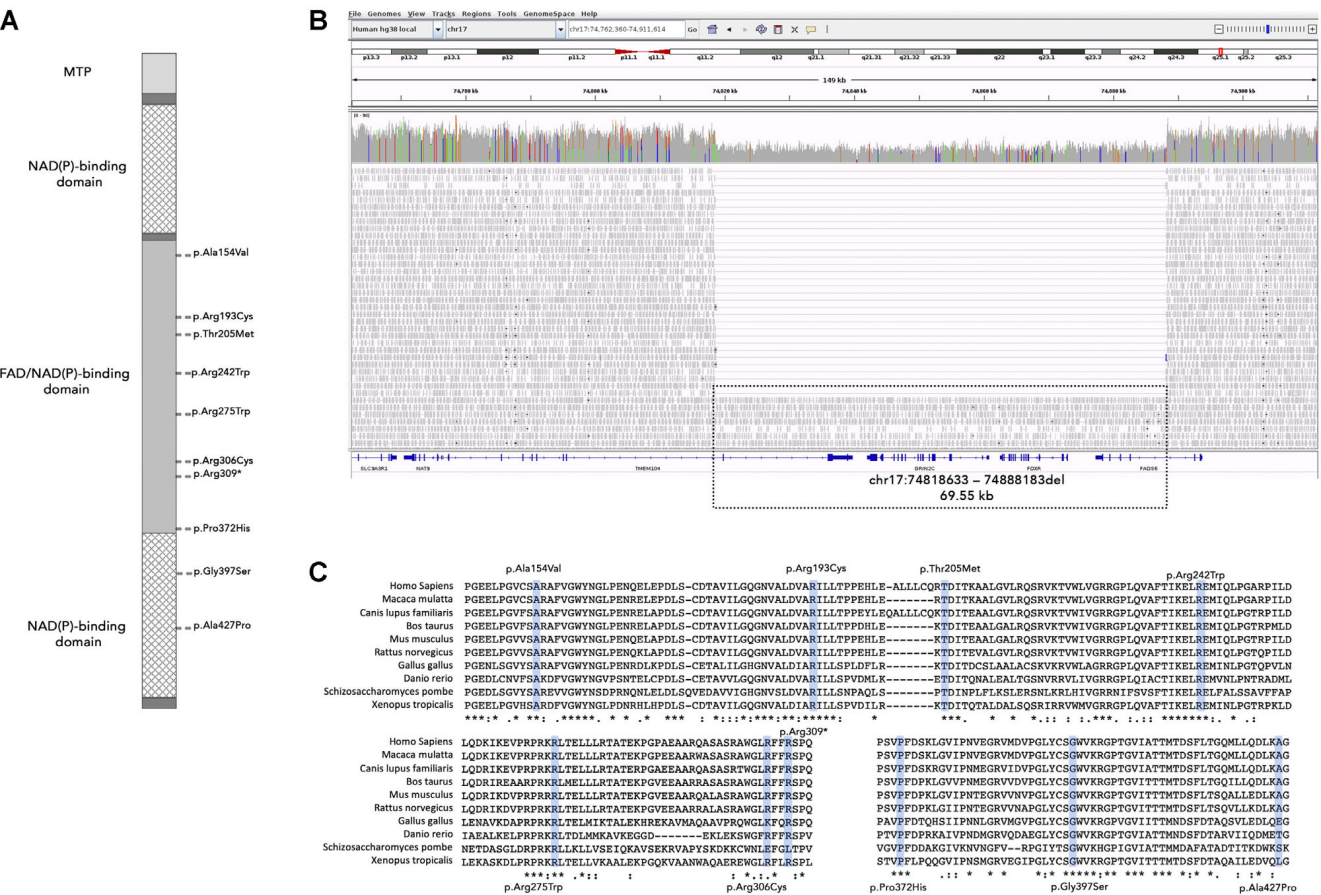
(**A**) Schematic diagram of the ferredoxin reductase protein. Domains are indicated by different shading. The identified variants are indicated on the right side of the bar at the corresponding positions. (**B**) A visualization of a large deletion spanning the two 3′ exons of *TNEM104*, *FDXR*, *GRIN2C*, and the four 3′ exons of *FADS6* identified in one individual from family 6 using the Integrative Genomics Viewer (IGV). (**C**) Multiple alignment of ferredoxin reductase orthologues. All mutated sites are strictly conserved in diverse species.

We identified 10 individuals from 8 unrelated families (Fig. 1) with clinical features of bilateral optic atrophy (*N* = 10), retinal dystrophy (*N* = 7), and extra-ocular findings consequent upon biallelic variants in the *FDXR* gene (Table 1, Fig. 2A). Half of the study subjects had sensorineural hearing loss, which had previously been reported in association with biallelic variants in *FDXR*. Two individuals from unrelated families (2/10) were suspected to have Guillain-Barre syndrome. A summary of ophthalmological and neurological phenotypic features of the affected individuals is provided in Table 2.

**Table 1.** *FDXR* Genotypes Identified in Our Cohort

Family	Affected	HGVs	HGVp	Segregation	gnomAD Version 3.1 (MAF)	Domain	In Silico: Polyphen-2, Mutation Taster	ClinVAr
Family 1(GC 21294)	1	c.577C>Tc.1115C>A	p.(Arg193Cys)p.(Pro372His)	Both variants present in affected;Paternal DNA unavailable;Maternal: p.(Arg193Cys)	0.0000065730.0001315	FAD/NAD(P) BDFAD/NAD(P) BD	Possibly damaging; disease causingProbably damaging; disease causing	––
Family 2(GC 17577)	2	c. 925C>Tc.1115C>A	p.(Arg309*)^1^p.(Pro372His)	Both variants present in affected;Paternal: p.(Arg309*)Maternal: p.(Pro372His)	0.000026280.00001315	FAD/NAD(P) BDFAD/NAD(P) BD	Disease causingPossibly damaging; disease causing	Likely pathogenic–
Family 3(GC 15689)	1	c.461C>Tc.823C>T	p.(Ala154Val)p.(Arg275Trp)	Both variants present in affected;Paternal DNA unavailable;Maternal: p.(Arg275Trp)	00	FAD/NAD(P) BDFAD/NAD(P) BD	Probably damaging; disease causingProbably damaging; disease causing	––
Family 4	1	c.1115C>Ac.1279G>C	p.(Pro372His)p.(Ala427Pro)	Both variants present in affected;Paternal: p.(Ala427Pro)Maternal DNA unavailable;	0.000013150	FAD/NAD(P) BDNAD(P) BD	Possibly damaging; disease causingBenign, polymorphism	––
Family 5(GC 28630)	1	c.614C>Tc.823C>T	p.(Thr205Met)p.(Arg275Trp)	Both variants present in affected;Sibling II:1: no variants present;Sibling II:2: p.(Thr205Met);Sibling II:3: no variants present;	0.000013140	FAD/NAD(P) BDFAD/NAD(P) BD	Probably damaging; disease causingProbably damaging; disease causing	––
Family 6(GC 28579)	1	c.1115C>A	p.(Pro372His)	No other DNA available	0.000013150	FAD/NAD(P) BDWhole gene deletion	Possibly damaging; disease causing	––
		(chr17:74818633 – 74888183del)						
Family 7(GC 28550)	2	c.1115C>Ac.1189G>A	p.(Pro372His)p.(Gly397Ser)	Both variants present in affected;Paternal DNA unavailable;Maternal: p.(Gly397Ser)	0.000013150.00003285	FAD/NAD(P) BDNAD(P) BD	Possibly damaging; disease causingProbably damaging; disease causing	––
Family 8	1	c.724C>Tc.916C>T	p.(Arg242Trp)^2^p.(Arg306Cys)^2^	Both variants present in affected;No other DNA available	0.0000065730.00004600	FAD/NAD(P) BDFAD/NAD(P) BD	Probably damaging; disease causingBenign, disease causing	–Likely pathogenic

All variants reported by using NM_024417.4 nomenclature.

FAD/NAD(P) BD - binding domain; NAD(P) BD.

**Table 2.** Phenotypic Features and Systemic Manifestation of Affected Individuals

	Family 1	Family 2		Family 3	Family 4	Family 5	Family 6	Family 7		Family 8
Sex	M	M	M	M	M	F	M	M	F	F
Age of initial presentation	3 y	4 y	14 y	7 y	22 y	40s	Childhood	2.5 years	3.5 y	42 y
***Ophthalmological features***										
Age of examination	11 y	11 y	14 y	26 y	25 y	45 y	7 y	26 y	10	42 y
Optic atrophy	Yes (3)	Yes (11)	Yes (14)	Yes (7)	Yes (25)	Yes (40s)	Yes (>7)	Yes (7)	Yes (3.5)	Yes (42)
Retinal dystrophy	No	Yes (4)	Yes (14)	Yes (7)	No	No	Yes (7)	Yes (7)	Yes (3.5)	Possible
Macula edema	No	Yes (5)	No	No	No	No	No	Yes (17)	No	No
Retinal vessels attenuation	No	Yes (11)	Yes (14)	Yes (8)	No	No	No	Yes (ND)	No	No
Cataract	No	Yes (11)	No	Yes (18)	No	No	Yes (17 to 18)	No	No	No
Nystagmus	Yes (3)	No	No	Yes (14)	No	No	Yes (>7)	No	No	No
Squint	Yes (3)	No	No	Yes (16)	No	No	No	No	No	No
Latest BCVA	HM	LP	0.06 and 0.1	HM and LP	CF and 6/36	6/36 and 6/24	ND	6/12 and 6/18	6/36	ND
Constricted visual field	No	Yes	Yes	Yes	No	No	ND	Yes	Yes	ND
Nyctalopia	No	Yes	No	Yes	No	No	ND	ND	ND	ND
***Hearing impairment***	No	Yes (11)	Yes (late teens)	Yes (20)	No	Yes (40s)	No	No	No	Yes (42)
***Neurological features***										
Ataxia/balance problems	Yes (11)	Yes (late teens)	Yes (late teens)	No	No	Yes (40s)	Yes (childhood)	Yes (2.5)	Yes (15)	Yes (42)
Sensory neuropathy	No	Yes (late teens)	Yes (late teens)	No	Yes (22)	Yes (40s)	Yes (childhood)	No	No	Yes (42)
Motor neuropathy	No	Yes (late teens)	Yes (late teens)	No	Yes (22)	Yes (40s)	No	No	No	Yes (42)
Dizziness	Yes (11)	No	No	Yes (late teens)	No	No	No	No	No	No
Tremor	Yes (12)	No	No	Yes (15)	No	No	No	No	No	No

Age of onset indicated in years in brackets.

F, female; M, male; BCVA, best corrected visual acuity; HM, hand motions; LP, light perception; CF, counting fingers; ND, no data.

### Clinical Data of Individuals Carrying Biallelic FDXR Variants

**Figure 3.**
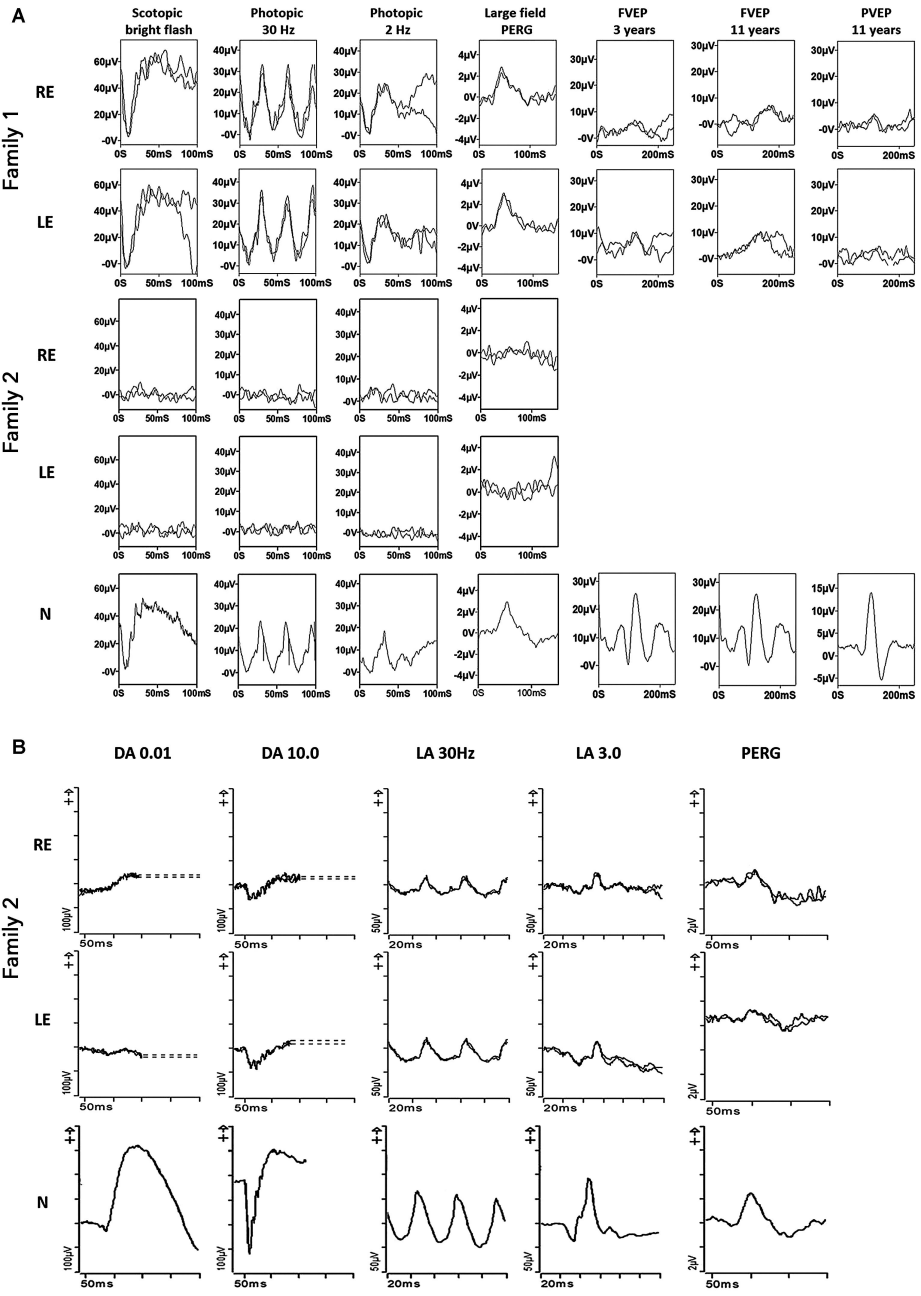
(**A**) – Electrophysiological recordings from right eye (RE) and left eye (LE) of the proband in family 1, the younger sibling from family 2 and from one eye of an unaffected control subject (N) for comparison. The flash ERGs and large field pattern ERGs were recorded using lower eyelid skin electrodes at the age of 3-years (proband family 1) or 5 years (younger sibling; family 2); flash VEPs (FVEP) were recorded in the 3-year-old and repeated with additional pattern reversal VEPs (PVEP) at the age of 11 years. The ERGs and PERG P50 component in the proband of family 1 are normal but the abnormal PERG N95:P50 ratio, abnormal PVEP and progressively abnormal flash VEPs are consistent with bilateral retinal ganglion cell/optic nerve dysfunction. The younger sibling in family 2 has undetectable ERGs and PERG, in keeping with severe rod and cone photoreceptor function with severe macular involvement. (**B**) International standard full-field ERGs and pattern ERGs from the right eye (RE) and left eye (LE) of the 14-year-old proband in family 2 and from an unaffected control subject (N) for comparison. The recordings were obtained using gold foil corneal electrodes and show ERG evidence of a rod-cone dystrophy with PERG P50 reduction consistent with relatively mild macular involvement.

The proband from family 1 presented with decreased vision of approximately 1.3 logMar in both eyes at the age of 3 years. He had rotary nystagmus in all directions of gaze, a left convergent squint, and small pale optic nerves bilaterally. He was otherwise fit and healthy with no neurological deficit or cerebellar dysfunction. At the age of 11 years, he complained of headaches and visual hallucinations, which were thought to be due to Charles-Bonnet syndrome. Around that time, he started reporting dizziness and balance issues that progressed over time. He developed shaking of his hands (tremor) at the age of 12 years. Neuroimaging showed slender optic nerves and chiasm with no other intracranial abnormalities. Scotopic and photopic flash ERGs at the age of 3 years revealed no evidence of generalized retinal dysfunction. The PERG P50 components were of short peak time and preserved amplitude but the N95:P50 ratios were reduced and flash VEPs subnormal, in keeping with bilateral retinal ganglion cell/optic nerve dysfunction. Pattern VEPs performed at the age of 11 years were delayed and subnormal and there was marked worsening of flash VEP timing, in keeping with progressive optic nerve dysfunction (Fig. 3A). Retinal imaging is not available. Previous genetic screening of the mitochondrial genome was negative.

**Figure 4.**
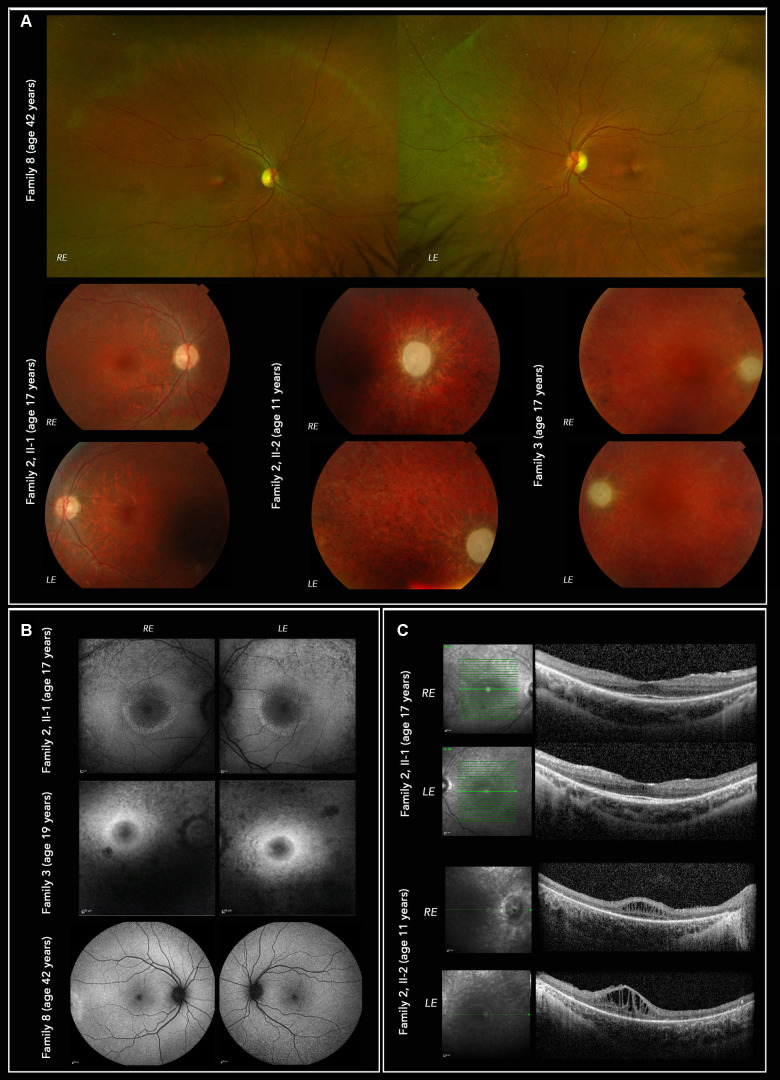
Multimodal imaging of affected individuals from family 2, family 3, and family 8. (**A**) Fundus color photographs, Optos wide-angle upper panels (family 8) show retinal pigmentary changes in the far periphery (nasal to the disc). Topcon low panels show optic atrophy and retinal vessel attenuation in family 2 II-1, II-2 and family 3. (**B**) Heidelberg Spectralis autofluorescence images (30 degree upper 4 panels, 55 degrees lower 2 panels). Both family 2 II-1 and family 3 show hyperautofluorescent rings (of Robson^36^) delineating preservation of the central, outer retinal structure (see **C**). (**C**) SD-OCT imaging of individuals from family 2 showing in family 2 II-1 preserved ellipsoid and outer nuclear layer only within the autofluorescent ring (see **B**). In family 2 II-2, there is greater loss of outer retina with some visible ellipsoid centrally, as well as significant macular oedema. The overall retinal thickness is significantly reduced.

The proband from family 2 was 14 years old when an abnormal retina appearance was noted during a routine optician consultation. At that time, he was asymptomatic with visual acuities of 0.06 logMar in the right eye and 0.1 logMar in the left eye. Visual field testing showed significantly constricted fields. Fundus examination revealed bilateral optic atrophy with atrophy and pigment migration in the mid periphery of the retina (Fig. 4A). Later, he developed mild sensorineural hearing loss and peripheral neuropathy. ISCEV-standard electrophysiology revealed ERG evidence of a rod-cone dystrophy and PERG P50 reduction in keeping with mild macular involvement; there was no evidence of a reduced PERG N95:P50 ratio (see Fig. 3B). His younger brother started experiencing symptoms at the age of 4 years with reduced vision under low light conditions. At 5 years, scotopic and photopic flash ERGs and PERG were undetectable, consistent with severe photoreceptor dysfunction with severe macular involvement (see Fig. 3A). At the age of 11 years, ophthalmological examination showed early cataracts, bilateral optic atrophy, retinal vessel attenuation, peripheral pigmentary changes (see Fig. 4A), and macular edema (see Fig. 4C, II-2), which developed at the age of 5 years and was unresponsive to treatment with oral acetazolamide. At the same age, he was diagnosed with profound hearing impairment. Similar to his older brother, he subsequently developed peripheral neuropathy. In addition, he was diagnosed with verbal dyspraxia.

The proband from family 3, now aged 27 years, was diagnosed with bilateral optic atrophy and retinal dystrophy at the age of 7 years. His best corrected visual acuities were 0.79 logMar in the right eye and 0.9 logMar in the left eye. Ophthalmological examination revealed pale optic nerves, atrophic retina, and a slight epiretinal membrane bilaterally. At the age of 8 years, flash ERGs showed evidence of marked retinal dysfunction, affecting both rods and cones. ISCEV-standard PERGs, of sufficient technical quality in the left eye only, were undetectable suggesting severe macular involvement. Pattern reversal VEPs were undetectable and flash VEPs delayed and subnormal bilaterally. Within a year, the retinal vessels were lost, followed by retinal pigmentary changes (see Fig. 4A). At the age of 14 years, he developed horizontal nystagmus in both eyes, which changed to pendular nystagmus in the next 2 years. At the age of 15 years, he was diagnosed with a tremor affecting his right upper limb, that progressed to involve both arms by the age of 17 years. The proband had an early cataract bilaterally diagnosed at the age of 18 years, progressing to a dense cataract with no fundus view in the left eye 6 years later. At the age of 20 years, he was diagnosed with sensorineural hearing loss. Neuroimaging was normal other than a posterior fossa arachnoid cyst that was an incidental finding. The patient had learning difficulties, in particular, delay in reading and writing that were thought to be secondary to the visual impairment.

The proband from family 4 developed acute neurological symptoms at the age of 22 years, which was thought to represent a mild form of Guillain-Barre syndrome. Within a year of presentation, he experienced a relapse and lower limb distal flaccid paresis with areflexia and peripheral dysesthesia. Neurophysiological studies at that time indicated a combined peripheral motor and sensory neuropathy involving the lower limbs. He underwent muscle biopsy, which showed evidence of very significant axonal loss likely secondary to the demyelination process. He was treated with intravenous immunoglobulins with no significant effect. At the age of 24 years, he experienced subacute severe reduced vision in both eyes associated with a significant increase in his peripheral dysesthesia. His visual acuity was counting fingers in the right eye and 0.6 logMar in the left eye. Fundus examination showed atrophic pale optic nerve head bilaterally. Visual electrophysiology testing revealed delayed VEPs bilaterally with more severe changes observed in the right eye. In addition, he became increasingly short of breath and a significant reduction in exercise tolerance. Neuroimaging did not identify any significant abnormality. As the clinical picture of visual loss resembled that of Leber hereditary optic neuropathy (LHON) presentation, and the neurological involvement suggested spinocerebellar ataxia, genetic testing for LHON and *PMP22* was performed, which did not identify any pathogenic variant. Retinal imaging is not available.

The proband from family 5 was born at term by normal delivery and met all milestones during development. However, she was noticed to have some learning difficulties at school. In additional, she was noted to be clumsy and there was a suggestion of mild deafness long before she suffered sudden onset of bilateral severe deafness in her 40s. The onset of hearing loss was followed by foot pain with pins and needles and deterioration of vision. On examination, she had evidence of a mild neuropathy, minimal weakness of ankle dorsiflexion, absent lower limb reflexes, and reduction of vibration to the costal margins bilaterally. Neuro-otological investigations showed bilateral symmetrical sensorineural hearing loss. Her visual acuity was 0.78 logMar in the right eye and 0.6 logMar in the left eye and ophthalmological investigations confirmed bilateral optic atrophy. Interestingly, VEPs from both eyes were well formed and of normal amplitude. However, the center field latencies were markedly increased and the whole field responses were at the upper limit of normal. The findings were suggestive of bilateral demyelinating optic neuropathy. Extensive investigations indicated a severe axonal length dependent polyneuropathy affecting mostly the lower limbs. A brain magnetic resonance imaging (MRI) scan was within normal limits; the optic nerves and optic chiasm appeared normal. Nerve conduction studies showed no demyelination. Electromyography showed active and chronic neurogenic changes of the right tibialis anterior and peroneus tertius. Sural nerve biopsy showed an axonal neuropathy with no evidence of primary demyelination. A muscle biopsy showed no specific features of mitochondrial disease. Respiratory chain analysis in muscle biopsy was normal and whole mitochondrial genome sequencing was normal. Over the years, she had developed a mild cerebellar syndrome and intermitted psychosis. In addition, she developed an episode of very severe hyperchloremic metabolic acidosis requiring hospitalization.

The proband from family 6 presented in infancy with ataxia due to poor coordination and balance. Throughout childhood he had three to four episodes of viral illness with fever, which would lead to him becoming more ataxic followed by recovery but not to his baseline. At the age of 7 years, he was diagnosed with retinitis pigmentosa. Later, he developed bilateral nystagmus and optic atrophy. Retinal imaging is not available. At around 17 to 18 years of age, he was found to have cataracts. In addition, he was diagnosed with attention deficit hyperactivity disorder (ADHD) and hypermobility. His mother and sister also have hypermobility. Previous genetic screening of the mitochondrial genome was negative.

The proband from family 7 presented with visual symptoms at the age of 3.5 years. Her visual acuity was 0.78 logMar in both eyes. She was diagnosed with optic atrophy and retinal dystrophy. At the age of 15 years, she developed evidence of posterior column disease with mild gait ataxia, brisk knee jerks, absent ankle jerks, and impaired vibration sense to the knee. In addition, she developed cyclical vomiting leading to investigation for a gastrointestinal tract disorder, which did not identify any abnormality. Genetic testing including for known mitochondrial mutations and for genes associated with ataxia were normal. Her brother developed neurological symptoms at age 2.5 years and was suspected to have Guillain-Barre syndrome. He underwent a muscle biopsy, which showed an increase in fiber size and variability in diameter with unclear clinical significance. He was subsequently diagnosed with optic atrophy and rod cone dystrophy. Retinal imaging is not available. At the age of 12 years, neurological examination revealed absent ankle jerks, impaired joint position, and vibration sense and gait ataxia. Similar to his sister, genetic testing was normal.

The proband from family 8 presented with neuropathic pain at the age of 42 years. Nerve conduction studies showed sensory neuropathy; she later developed an additional motor neuropathy and subsequent sensory ataxia and motor weakness. Ophthalmic examination showed optic atrophy, which was confirmed by optical coherence tomography (OCT) imaging. Color fundus imaging showed retinal pigmentary changes in the far periphery nasal to the disc (see Fig. 4A) with outer retinal thinning at the macula suggestive of retinal degeneration. In addition, she had hearing loss with additional signs and symptoms, namely vomiting, constipation, and severe weight loss, suggesting gastrointestinal tract involvement.

### Molecular Genetic Analysis

Eleven distinct candidate *FDXR* variants were identified in 10 affected individuals from 8 families (see Table 1). Nine (81.8%) of these were missense variants with only one stop-gain and one entire gene deletion observed. No individuals had a biallelic null genotype. A large deletion (chr17:74818633–74888183del) spanning the two 3′ exons of *TNEM104*, *FDXR*, *GRIN2C*, and the four 3′ exons of *FADS6*, was identified in one individual from family 6 in the heterozygous state (see Fig. 2B). To determine if any of the additional affected genes could be a candidate for retinal dystrophy, optic atrophy, or ataxia, we reviewed the Online Mendelian Inheritance in Man (OMIM) database, literature, and gnomAD constraint data revealing no compelling evidence for causality. This individual also harbored a previously unreported variant (NM_024417.4: c.1115C>A, p.(Pro372His)) in the hemizygous state falling within the region deleted on the trans allele (demonstrated by reviewing individual reads using the Intergrative Genomics Viewer [IGV]^15^ see Fig. 2B).

This variant was identified in seven affected individuals in five of eight (62.5%) families (see Table 1). This variant is present in 2 of 68,025 European alleles (MAF 0.00003) in gnomAD. Haplotype analysis of the 5 unrelated families showed a likely ancestral haplotype spanning 128kb around the *FDXR* gene (chr17:74819055 [rs35292847] to chr17:74947323 [rs34805215]) that was observed in all carriers from families 2, 6, and 7 (see Table 3). Recombinations were identified centromeric to chr17:74863955 in family 1 and telomeric to chr17:74863955 in family 4 (Table 3). All genotypes were confirmed to be biallelic with the exception of that in family 8 where no segregation was possible.

**Table 3.** Haplotype Reconstruction for Individuals Carrying c.1115C>A, p.(Pro372His) Variant

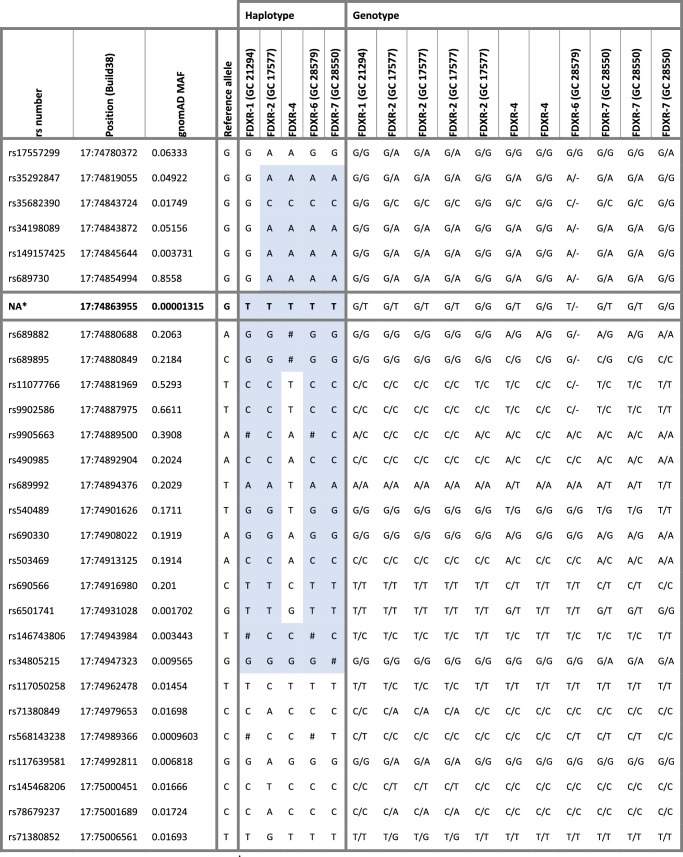

Haplotypes were reconstructed using WGS datasets (NIHR-RD and 100KGP study).

* c.1115C>A, p.(Pro372His) variant. # undetermined single nucleotide polymorphism (SNP). Shaded haplotype is shared. Underlined individuals are c.1115C>A, p.(Pro372His) variant carriers.

The majority of coding *FDXR* variants (8/10) were located within the FAD/NAD(P) binding domain with two remaining variants located in the NAD(P) binding domain (see Fig. 2A). All candidate missense *FDXR* variants were predicted to be damaging/disease causing by in silico prediction algorithms and rare or absent from the gnomAD dataset. Multiple alignment of FDXR orthologues confirmed the strict conservation of the protein across different species with complete conservation of all affected amino acid residues in mammalian orthologues (see Fig. 2C). ACMG classification^16^ for each variant is provided in Supplementary Table S2.

## Discussion

We report the detailed clinical and genetic findings in a series of 10 individuals from 8 unrelated families affected by *FDXR-*associated disease. To date, biallelic variants in *FDXR* have been reported to cause mitochondrial syndromic optic atrophy and sensorineural hearing loss. Thirty-five cases are reported in the literature with a variable phenotype featuring optic atrophy as a unifying clinical finding observed in all individuals.^1^^–^^5^ It is not unusual to observe ocular manifestations in mitochondrial disease, which can occur as isolated optic atrophy or accompanied by additional systemic abnormalities. Interestingly, *FDXR*-associated optic neuropathy seems to be variable and may present similar to *OPA1*-associated optic atrophy, Wolfram syndrome, or LHON. However, retinal dystrophy is not observed in these disorders. Extra-ocular findings, such as the severe neurological manifestations seen in *FDXR*-disease, are not typical for these well-characterized optic neuropathies, whereas diabetes, which is classically seen in autosomal recessive Wolfram syndrome and maternally inherited diabetes deafness (MIDD), has not been observed in *FDXR*-associated disease to date.

Here, we report retinal dystrophy in 7 of 10 individuals with no apparent clustering of genotypes in the remaining 3 affected individuals. Optic atrophy is a major manifestation of mitochondrial disease and, with pigmentary retinopathy, is being increasingly recognized in patients with both mutations in mitochondrial DNA and nuclear-encoded mitochondrial proteins.^17^^–^^22^ The pigmentary retinal changes can be mild and they are frequently missed in the absence of a detailed fundus examination and more sophisticated retinal fundus autofluorescence imaging. Multimodal imaging is therefore useful to fully characterize any retinal involvement in patients with confirmed or suspected mitochondrial diseases, including *FDXR*-associated disease. Furthermore, visual electrophysiology is an essential test that can provide additional evidence of an underlying photoreceptor dysfunction in addition to retinal ganglion cells and optic nerve dysfunction, when clinical examination alone does not detect retinal degeneration. Although a retinal dystrophy has been described in 7 of 34 (20.6%) previously reported cases of *FDXR-*associated disease, there is limited information on the retinal pathology (Supplementary Table S3) and the findings were not highlighted as part of the disease spectrum.

The mechanisms of outer retinal degeneration in *FDXR-*associated disease have not yet been clarified, although, in the current study, retinal imaging and electrophysiology has demonstrated that the site of pathology is likely to be the photoreceptor. Mitochondria plays a key role in the assembly and biogenesis of mitochondrial iron-sulfur clusters, which is essential for the maturation of not only mitochondrial iron-sulfur proteins, but also those required for cytosolic and nuclear functions.^6^^,^^7^^,^^23^ Iron-sulfur clusters are ubiquitous enzymatic cofactors essential for multiple cellular processes, both inside and outside the mitochondria, including participation in mitochondrial respiration and regulation of iron metabolism. It is well-established that ferredoxin reductase is necessary for the biogenesis of these clusters and pathogenic variants affecting proteins driving the multistep biosynthesis and maturation pathway disrupt iron homeostasis within the cell and cause severe metabolic, systemic, neurological and hematological disorders.^6^^,^^7^^,^^23^ Ferredoxin reductase deficiency has been reported to cause mitochondrial disease with broad spectrum of phenotypic features dominated by nervous system involvement. Functional assays in patient fibroblasts and tissues from a murine *Fdxr* mutant model have shown multiple biochemical and metabolic consequences, including reduction of electron transport due to impaired activity of the mitochondrial respiratory chain complexes, reduced ATP production, and a significant increase in the production of reactive oxygen species (ROS).^4^^,^^6^^,^^23^^,^^24^ The production of ROS is a normal metabolic process and ROS accumulation in the retina is an important mechanism leading to cell death and disease.^25^ Recent functional studies demonstrated that *FDXR* variants lead to a partial impairment of the electron transport along the mitochondrial respiratory chain and elevation of ROS.^4^^,^^24^ Iron excess has been reported within mitochondria with depolarization of the mitochondrial membrane in mutant *Fdxr* mice, highlighting the role of ferredoxin reductase in iron homeostasis.^24^ Furthermore, abnormal retinal iron metabolism may lead to a variety of retinal changes, including the ones observed in hereditary iron overload disorders namely, aceruloplasminemia, pantothenate kinase associated neurodegeneration, Friedreich's ataxia (in which there is progressive iron accumulation within mitochondria), and those observed in the current study.^26^^–^^28^ Interestingly, variants in *FDX2*, encoding another mitochondrial ferredoxin (FDX2) have been associated with mitochondrial disease, including optic atrophy but without retinal involvement.^29^^,^^30^ However, the present study and data from other iron overload disorders suggest ophthalmological assessment, including multimodal imaging, may be advised in such cases. Single-cell RNAseq data suggests that the average expression of *FDXR* within the human neural retina is relatively low, for example, compared with *SSBP1* (see Supplementary Table S4 for graphical illustration of both genes RNAseq data), defects of which similarly cause both optic atrophy and retinal degeneration.^17^^–^^19^^,^^31^

Thus, *FDXR*-associated disease may be a multifactorial disorder precipitated by mitochondrial dysfunction, accumulation of ROS, iron-induced oxidative damage, defects in iron-sulfur clusters biogenesis, and possibly iron toxicity. On this basis, we hypothesize that the outer retinal changes observed within this cohort are secondary, due to a cumulative effect of these mechanisms rather than a primary photoreceptor dysfunction.^32^^,^^33^ Understanding the pathogenic mechanism could lead to a potential therapeutic strategy, for example, iron chelating agents have been trialed in the iron accumulation disorder, Freidrich's ataxia, or Idebenone, now with European Medical Association approval for LHON treatment.^34^^,^^35^

Interestingly, the murine *Fdxr* mutant demonstrated a significantly thinned retina mainly due to ganglion cell complex loss and the outer retina did not appear to be affected. In humans, optic atrophy has been documented in all cases with the retinal dystrophy reported in 7 of 34 cases.^1^^–^^4^ Thus, the retinal dystrophy and photoreceptor loss in this cohort may follow the primary retinal ganglion cell disease. Furthermore, *FDXR*-associated disease is in keeping with the marked variability seen in patients with mitochondrial disorders.

This study identified 7 individuals from 5 families harboring the previously unreported missense variant c.1115C>A, which is rare in the gnomAD dataset with only two alleles out of 68,000 demonstrating that it is enriched in the optic atrophy and retinal degeneration patient population. We demonstrate a likely ancestral haplotype harboring this variant with no evidence to suggest it has arisen independently in different families. This may therefore represent a significant cause of *FDXR*-disease in the British population.

Overall, we report retinal dystrophy as a major clinical feature observed in patients harboring biallelic *FDXR* variants. In addition, our observation suggests that multiple factors, such as mitochondrial dysfunction, accumulation of ROS and iron could lead to the characteristic combination of optic atrophy and retinal degeneration.

## Supplementary Material

Supplement 1

Supplement 2

Supplement 3

Supplement 4

## References

[1] Peng Y, Shinde DN, Valencia CA, et al. Biallelic mutations in the ferredoxin reductase gene cause novel mitochondriopathy with optic atrophy. *Hum Mol Genet**.* 2017; 26(24): 4937–4950. 29040572 10.1093/hmg/ddx377 PMC5886230

[2] Paul A, Drecourt A, Petit F, et al. FDXR mutations cause sensorial neuropathies and expand the spectrum of mitochondrial Fe-S-synthesis diseases. *Am J Hum Genet**.* 2017; 101(4): 630–637. 28965846 10.1016/j.ajhg.2017.09.007 PMC5630197

[3] Stenton SL, Piekutowska-Abramczuk D, Kulterer L, et al. Expanding the clinical and genetic spectrum of FDXR deficiency by functional validation of variants of uncertain significance. *Hum Mutat**.* 2021; 42(3): 310–319. 33348459 10.1002/humu.24160

[4] Slone J, Peng Y, Chamberlin A, et al. Biallelic mutations in FDXR cause neurodegeneration associated with inflammation. *J Hum Genet**.* 2019; 63(12): 1211–1222. 10.1038/s10038-018-0515-y PMC6451867 30250212

[5] Liu P, Meng L, Normand EA, et al. Reanalysis of clinical exome sequencing data. *N Engl J Med**.* 2019; 380(25): 2478–2480. 31216405 10.1056/NEJMc1812033 PMC6934160

[6] Rouault TA. Biogenesis of iron-sulfur clusters in mammalian cells: new insights and relevance to human disease. *Dis Model Mech**.* 2012; 5(2): 155–164. 22382365 10.1242/dmm.009019 PMC3291637

[7] Wachnowsky C, Fidai I, Cowan JA. Iron-sulfur cluster biosynthesis and trafficking - impact on human disease conditions. *Metallomics*. 2018; 10(1): 9–29. 29019354 10.1039/c7mt00180k PMC5783746

[8] Bach M, Brigell MG, Hawlina M, et al. ISCEV standard for clinical pattern electroretinography (PERG): 2012 update. *Doc Ophthalmol**.* 2013; 126(1): 1–7. 23073702 10.1007/s10633-012-9353-y

[9] McCulloch DL, Marmor MF, Brigell MG, et al. ISCEV Standard for full-field clinical electroretinography (2015 update). *Doc Ophthalmol**.* 2015; 130(1): 1–12. 10.1007/s10633-014-9473-7 25502644

[10] Holder GE, Robson AG. Paediatric electrophysiology: a practical approach. In: *Essentials in Ophthalmology*. Lorenz B, ed. Berlin, Germany: Springer-Verlag; 2006: 133–155.

[11] Odom JV, Bach M, Brigell M, et al. ISCEV standard for clinical visual evoked potentials: (2016 update). *Doc Ophthalmol**.* 2016; 133(1): 1–9. 10.1007/s10633-016-9553-y 27443562

[12] Carss K, Arno G, Erwood M, et al. Comprehensive rare variant analysis via whole-genome sequencing to determine the molecular pathology of inherited retinal disease. *Am J Hum Genet**.* 2017; 100(1): 75–90. 28041643 10.1016/j.ajhg.2016.12.003 PMC5223092

[13] Taylor RL, Arno G, Poulter JA, et al. Association of steroid 5α-reductase type 3 congenital disorder of glycosylation with early-onset retinal dystrophy. *JAMA Ophthalmol**.* 2017; 135(4): 339. 28253385 10.1001/jamaophthalmol.2017.0046

[14] Martin AR, Williams E, Foulger RE, et al. PanelApp crowdsources expert knowledge to establish consensus diagnostic gene panels. *Nat Genet**.* 2019; 51(11): 1560–1565. 31676867 10.1038/s41588-019-0528-2

[15] Thorvaldsdóttir H, Robinson JT, Mesirov JP. Integrative genomics viewer (IGV): high-performance genomics data visualization and exploration. *Brief Bioinform*. 2013; 14(2): 178–192. 22517427 10.1093/bib/bbs017 PMC3603213

[16] Richards S, Aziz N, Bale S, et al. Standards and guidelines for the interpretation of sequence variants: a joint consensus recommendation of the American College of Medical Genetics and Genomics and the Association for Molecular Pathology. *Genet Med**.* 2015; 17(5): 405–424. 25741868 10.1038/gim.2015.30 PMC4544753

[17] Del Dotto V, T Pippucci, Carelli V, et al. SSBP1 mutations cause mtDNA depletion underlying a complex optic atrophy disorder. *J Clin Invest**.* 2020; 130(1): 108–125. 31550240 10.1172/JCI128514 PMC6934201

[18] Piro-mégy C, Solà M, Delettre C. Dominant mutations in mtDNA maintenance gene SSBP1 cause optic atrophy and foveopathy. *J Clin Invest**.* 2020; 130(1): 143–156. 31550237 10.1172/JCI128513 PMC6934222

[19] Jurkute N, Leu C, Pogoda H, et al. SSBP1 mutations in dominant optic atrophy with variable retinal degeneration. *Ann Neurol**.* 2019; 86(3): 368–383. 31298765 10.1002/ana.25550 PMC8855788

[20] Spiegel R, Pines O, Ta-Shma A, et al. Infantile cerebellar-retinal degeneration associated with a mutation in mitochondrial aconitase, ACO2. *Am J Hum Genet**.* 2012; 90(3): 518–523. 22405087 10.1016/j.ajhg.2012.01.009 PMC3309186

[21] Oh JK, de Carvalho JRL, Nuzbrokh Y, et al. Retinal manifestations of mitochondrial oxidative phosphorylation disorders. *Investig Ophthalmol Vis Sci*. 2020; 61(12): 12. 10.1167/iovs.61.12.12 PMC7571321 33049060

[22] Rajabian F, Manitto MP, Palombo F, et al. Combined optic atrophy and rod–cone dystrophy expands the RTN4IP1 (optic atrophy 10) phenotype [published online ahead of print October 27, 2020]. *J Neuroophthalmol*, 10.1097/WNO.0000000000001124. 33136666

[23] Shi Y, Ghosh M, Kovtunovych G, et al. Both human ferredoxins 1 and 2 and ferredoxin reductase are important for iron-sulfur cluster biogenesis. *Biochim Biophys Acta**.* 2012; 1823(2): 484–492. 22101253 10.1016/j.bbamcr.2011.11.002 PMC3546607

[24] Slone JD, Yang L, Peng Y, et al. Integrated analysis of the molecular pathogenesis of FDXR-associated disease. *Cell Death Dis**.* 2020; 11(6): 423. 32499495 10.1038/s41419-020-2637-3 PMC7272433

[25] Rohowetz LJ, Kraus JG, Koulen P. Reactive oxygen species-mediated damage of retinal neurons: drug development targets for therapies of chronic neurodegeneration of the retina. *Int J Mol Sci**.* 2018; 19(11): 3362. 10.3390/ijms19113362 PMC6274960 30373222

[26] Yamaguchi K, Takahashi S, Kawanami T, et al. Retinal degeneration in hereditary ceruloplasmin deficiency. *Ophthalmologica* 1998; 212(1): 11–14. 9438577 10.1159/000027251

[27] Dunaief JL, Richa C, Franks EP, et al. Macular degeneration in a patient with aceruloplasminemia, a disease associated with retinal iron overload. *Ophthalmology**.* 2005; 112(6): 1062–1065. 15882908 10.1016/j.ophtha.2004.12.029

[28] Porter N, Downes SM, Fratter C, et al. Catastrophic visual loss in a patient with Friedreich ataxia. *Arch Ophthalmol**.* 2007; 125(2): 273–274. 17296906 10.1001/archopht.125.2.273

[29] Gurgel-Giannetti J, Lynch DS, Brandão de Paiva AR, et al. A novel complex neurological phenotype due to a homozygous mutation in FDX2. *Brain*. 2018; 141(8): 2289–2298. 30010796 10.1093/brain/awy172 PMC6061701

[30] Spiegel R, Saada A, Halvardson J, et al. Deleterious mutation in FDX1L gene is associated with a novel mitochondrial muscle myopathy. *Eur J Hum Genet**.* 2014; 22(7): 902–906. 24281368 10.1038/ejhg.2013.269 PMC4060119

[31] Lukowski SW, Lo CY, Sharov AA, et al. A single-cell transcriptome atlas of the adult human retina. *EMBO J**.* 2019; 38(18): e100811. 31436334 10.15252/embj.2018100811 PMC6745503

[32] Loh A, Hadziahmetovic M, Dunaief JL. Iron homeostasis and eye disease. *Biochim Biophys Acta**.* 2009; 1790(7): 637–649. 19059309 10.1016/j.bbagen.2008.11.001 PMC2718721

[33] He X, Hahn P, Iacovelli J, et al. Iron homeostasis and toxicity in retinal degeneration. *Prog Retin Eye Res**.* 2007; 26(6): 649–673. 17921041 10.1016/j.preteyeres.2007.07.004 PMC2093950

[34] Zesiewicz TA, Hancock J, Ghanekar SD, et al. Emerging therapies in Friedreich's ataxia. *Expert Rev Neurother**.* 2020; 20(12): 1215–1228. 32909841 10.1080/14737175.2020.1821654 PMC8018609

[35] Carelli V, Carbonelli M, de Coo IF, et al. International consensus statement on the clinical and therapeutic management of leber hereditary optic neuropathy. *J Neuroophthalmol*. 2017; 37(4): 371–381. 28991104 10.1097/WNO.0000000000000570

[36] Robson AG, Saihan Z, Jenkins SA, et al. Functional characterisation and serial imaging of abnormal fundus autofluorescence in patients with retinitis pigmentosa and normal visual acuity. *Br J Ophthalmol**.* 2006; 90(4): 472–479. 16547330 10.1136/bjo.2005.082487 PMC1856999

