# Supplementary material for: Expanding the FDXR-Associated Disease Phenotype: Retinal Dystrophy Is a Recurrent Ocular Feature
Source: Invest Ophthalmol Vis Sci. 2021 May 3;62(6):2. doi: 10.1167/iovs.62.6.2 (PMC8107637; doi:10.1167/iovs.62.6.2)
Supplement: Supplement 1 [file iovs-62-6-2_s001.pdf]

Supplementary Table 1. Additional rare (MAF<0.001) variants (optic neuropathy and retinal dystrophy gene panels, PanelApp) identified in affected individuals.

Family	***Position (Build38)***	***Symbol***	***HGVSc***	***HGVSp***	***gnomAD***	***GT***	***Effect***	***Excluded***
Family-1 (GC 21294)	1:12064940C>T	*MFN2*	ENST00000235329.5:c.1451C>T	p.Thr484Met	5.80847E-05	0/1	missense	too frequent for dominant variant
	11:86662356A>C	*FZD4*	ENST00000531380.1:c.1442T>G	p.Ile481Ser	0	0/1	missense	unrelated to phenotype
	6:80634687A>T	*ELOVL4*	ENST00000369816.4:c.351T>A	p.Asn117Lys	0.000249198	0/1	missense	unrelated to phenotype
	8:68089945T>C	*CSPP1*	ENST00000262210.5:c.3125T>C	p.Val1042Ala	2.52684E-05	0/1	missense	unrelated to phenotype /heterozygous only
Family-2 (GC 17577)	16:49672770G>C	*ZNF423*	ENST00000262383.2:c.293C>G	p.Pro98Arg	0.000265223	0/1	missense	unrelated to phenotype
	16:72130111G>A	*DHX38*	ENST00000268482.3:c.55G>A	p.Asp19Asn	0.000787994	0/1	missense	unrelated to phenotype /heterozygous only
	17:6331726A>T	*AIPL1*	ENST00000381129.3:c.377T>A	p.Met126Lys	5.70125E-05	0/1	missense	unrelated to phenotype /heterozygous only
	5:89979523G>T	*GPR98*	ENST00000405460.2:c.5785G>T	p.Ala1929Ser	0.000679299	0/1	missense	unrelated to phenotype /heterozygous only
	7:21599223C>T	*DNAH11*	ENST00000328843.6:c.695C>T	p.Pro232Leu	0.000382072	0/1	missense	unrelated to phenotype /heterozygous only
	9:2718887G>T	*KCNV2*	ENST00000382082.3:c.1148G>T	p.Arg383Leu	0.000340732	0/1	missense	unrelated to phenotype /heterozygous only
Family-3 (GC 15689)	12:88089033delT	*CEP290*	ENST00000552810:c.4028del	p.Lys1343ArgfsTer2	0	0/1	frameshift	heterozygous only
	21:45468430C>T	*COL18A1*	ENST00000359759:c.1540C>T	p.Arg514Trp	0.00076	0/1	missense	unrelated to phenotype/heterozygous only
	5:83545656A>T	*VCAN*	ENST00000265077:c.9379+6A>T	-	0.00001	0/1	splice region	unrelated to phenotype/heterozygous only
Family 4	none							
Family 5 (GC 28630)	4:625846C>T	*PDE6B*	ENST00000496514:c.220C>T	p.Arg74Cys	0.00062	0/1	missense	heterozygous only
	2:111994390T>A	*MERTK*	ENST00000295408:c.1436T>A	p.Phe479Tyr	0.00006	0/1	missense	heterozygous only
Family 6 (GC 28579)	m:3892A>G	*MT-ND1*	ENST00000361390.2:c.586A>G	p.Thr196Ala	0.0002481	homo	missense	reported as benign
	6:152206274C>T	*SYNE2*	ENST00000367255.10:c.22913G>A	p.Gly7638Asp	0.0003876	0/1	missense	reported as benign/not related to phenotype
	6:152353329C>A	*SYNE2*	ENST00000367255.10:c.11187G>T	p.Lys3729Asn	0.004042	0/1	missense	reported as benign/not related to phenotype/too frequent
Family 7 (GC 28550)	m:8238T>C	*MT-CO2*	ENST00000361739.1:c.653T>C	p.Ile218Thr	0.0001063	homo	missense	scored as likely benign
Family 8	NA							

GT-genotype, NA – not available.
