# Supplementary material for: Expanding the FDXR-Associated Disease Phenotype: Retinal Dystrophy Is a Recurrent Ocular Feature
Source: Invest Ophthalmol Vis Sci. 2021 May 3;62(6):2. doi: 10.1167/iovs.62.6.2 (PMC8107637; doi:10.1167/iovs.62.6.2)
Supplement: Supplement 2 [file iovs-62-6-2_s002.pdf]

Supplementary Table 2. ACMG classification for each variant identified in affected individuals.

Family	*Affected*	*HGVs*	*HGVp*	*ACMG classification*[16]
Family-1 (GC 21294)	1	c.577C>T	p.(Arg193Cys)	VUS (PM2 moderate, PP2 supporting, PP3 supporting)
		c.1115C>A	p.(Pro372His)	Likely pathogenic (PS4 supporting, PM2 moderate, PP2 supporting, PP3 supporting)
Family-2 (GC 17577)	2	c. 925C>T	p.(Arg309*)[1]	Pathogenic (PVS1 very strong, PM2 moderate, PP3 supporting, PP5 supporting)
		c.1115C>A	p.(Pro372His)	Likely pathogenic (PS4 supporting, PM2 moderate, PM3 moderate, PP2 supporting, PP3 supporting)
Family-3 (GC 15689)	1	c.461C>T	p.(Ala154Val)	VUS (PM2 moderate, PP2 supporting, PP3 supporting)
		c.823C>T	p.(Arg275Trp)	VUS (PM2 moderate, PP2 supporting, PP3 supporting)
Family-4	1	c.1115C>A	p.(Pro372His)	Likely pathogenic (PS4 supporting, PM2 moderate, PP2 supporting, PP3 supporting)
		c.1279G>C	p.(Ala427Pro)	VUS (PM2 moderate, PP2 supporting, BP4 supporting)
Family -5 (GC 28630)	1	c.614C>T	p.(Thr205Met)	VUS (PM2 moderate, PP2 supporting, PP3 supporting)
		c.823C>T	p.(Arg275Trp)	VUS (PM2 moderate, PP2 supporting, PP3 supporting)
Family-6 (GC 28579)	1	c.1115C>A	p.(Pro372His)	Likely pathogenic (PS4 supporting, PM2 moderate, PP2 supporting, PP3 supporting)
		(chr17:74818633 – 74888183del)		NA
Family-7 (GC 28550)	2	c.1115C>A	p.(Pro372His)	Likely pathogenic (PS4 supporting, PM2 moderate, PP2 supporting, PP3 supporting)
		c.1189G>A	p.(Gly397Ser)	VUS (PM2 moderate, PP2 supporting, PP3 supporting)
Family-8	1	c.724C>T	p.(Arg242Trp)[2]	Pathogenic (PS3 strong, PM2 moderate, PM3 moderate, PP1 supporting, PP2 supporting, PP3 supporting)
		c.916C>T	p.(Arg306Cys)[2]	Pathogenic (PS3 strong, PM2 moderate, PP1 supporting, PP2 supporting, PP3 supporting, PP5 supporting)
