# Supplementary material for: Expanding the FDXR-Associated Disease Phenotype: Retinal Dystrophy Is a Recurrent Ocular Feature
Source: Invest Ophthalmol Vis Sci. 2021 May 3;62(6):2. doi: 10.1167/iovs.62.6.2 (PMC8107637; doi:10.1167/iovs.62.6.2)
Supplement: Supplement 3 [file iovs-62-6-2_s003.pdf]

Supplementary table 3. Retinal changes mentioned in previous reported cases.

Family (original)	Transcript	Genotype	Details	Reference
F2	NM_004110	c.1327G>A, p.Gly443Ser<br>c.151T>C, p.Phe51Leu	Leber's congenital amaurosis diagnosis at the age of 4 years. Later, at the age of 7 years, received a diagnosis of bilateral retinitis pigmentosa (full-field ERG revealed significantly delayed rod response with 70-92% reduction in amplitude).	*Peng et al. (Supplementary)*
F9	NM_004110	c.472G>A, p. Val158Met<br>c.637A>T, p.Ile213Phe	At the age of 3.5 years was diagnosed with retinitis pigmentosa. At that time, ERG revealed a diffuse cone-rod dystrophy. Longitudinal data showed gradual deterioration in vision. The patient gradually developed optic nerve atrophy, attenuation of the retinal vessels and pigmentary retinopathy.<br>OCT imaging was provided in supplementary material.	*Peng et al. (Supplementary)*
F10	NM_004110	c.944C>T, p.Arg315*<br>c.1226C>T, p.Pro409Leu	Retinopathy with loss of vision (no further details).	*Peng et al. (Supplementary)*
F11	NM_004110	c.1174C>T, p.Arg392Trp	Leber's congenital amaurosis diagnosis at the age of 22 months (no further details).	*Peng et al. (Supplementary)*
F12	NM_004110	c.472G>A, p. Val158Met<br>c.838A>T, p.Lys280*	Pigmentary retinopathy (proband). Brother was mentioned to have had retinitis pigmentosa (no further details).	*Peng et al. (Supplementary)*
F3	NM_024417	c.724C>T, p.Arg242Trp<br>c.979C>A, p.Arg327Ser	Retinitis pigmentosa diagnosis at the age of 2 years. ERG traces were absent. (No further details).	*Paul at al. (Supplementary)*
F6	NM_024417	c.1156C>T, p.Arg386Trp hom.	Retinal dystrophy (no further details).	*Stenton et al. (Table 2)*
